# Electronic Cigarettes for Curbing the Tobacco-Induced Burden of Noncommunicable Diseases: Evidence Revisited with Emphasis on Challenges in Sub-Saharan Africa

**DOI:** 10.1155/2016/4894352

**Published:** 2016-12-25

**Authors:** Jobert Richie N. Nansseu, Jean Joel R. Bigna

**Affiliations:** ^1^Department of Public Health, Faculty of Medicine and Biomedical Sciences, University of Yaoundé 1, Yaoundé, Cameroon; ^2^Sickle Cell Disease Unit, Mother and Child Centre of the Chantal BIYA Foundation, Yaoundé, Cameroon; ^3^Department of Epidemiology and Public Health, Centre Pasteur of Cameroon, Yaoundé, Cameroon; ^4^Faculty of Medicine, University of Paris Sud XI, Le Kremlin Bicêtre, France

## Abstract

*Introduction*. This review examines whether electronic cigarettes (e-cigs) implementation or vulgarization in sub-Saharan Africa (SSA) could be helpful in curtailing the toll of tobacco smoking in the region.* Discussion*. There are about 1.3 billion smokers worldwide, with nearly 80% of them living in developing countries where the burden of tobacco-related illnesses and deaths is the heaviest. Studies report that e-cigs may facilitate smoking cessation, reduction, or abstinence and may pose only a small fraction of the risks of traditional tobacco cigarettes; e-cigs may also considerably reduce second-hand smoking. Thereby, implementation of e-cig use could help to substantially reduce the burden driven by tobacco smoking in SSA, in a particular context of lack of regulations and control policies towards this threat. However, the evidence is not clear on whether e-cigs are risk-free, especially if used in the long term.* Conclusions*. On the whole, if e-cigs were to be introduced in SSA, they should be strictly recommended to current and/or ex-smokers as a method to quit smoking or prevent relapse and never-smokers should be strongly encouraged to avoid using these devices. Bans on sales of e-cigs to youngsters should be legislated, e-cig advertisements prohibited, and their usage continuously controlled and monitored.

## 1. Background

Currently, tobacco smoking is among the leading causes of preventable deaths globally, and its burden has been continuously increasing over the recent decades. Indeed, smoking increased from third to second among the global risk factors for disability adjusted life deaths (DALYs) between 1990 and 2013 [1]. It is estimated to be responsible for around 6.1 million deaths annually and 143.5 million DALYs, with over 600,000 deaths due to exposure to second-hand smoke [1, 2]. Yet, if nothing is done, the annual toll due to tobacco smoking is projected to increase up to 8 million deaths per year by 2030 [2]. Moreover, tobacco use increases the risk of cardiovascular disease (CVD), chronic respiratory disease, diabetes, cancer, and premature death [1, 3–8].

The World Health Organization (WHO) has stated as the fifth global target to curb the burden of noncommunicable diseases (NCDs) by 2025 “a 30% relative reduction in the prevalence of current tobacco use in persons aged 15+ years” [2]. Accordingly, WHO developed 6 MPOWER measures to ensure reduction in tobacco use by monitoring tobacco use and prevention policies (M), protecting people from second-hand smoke through national “100% smoke-free” legislation (P), offering help in quitting tobacco use (O), warning people about the dangers of tobacco use (W), enforcing bans on tobacco advertising, promotion, and sponsorship (E), and raising tobacco taxes (R) [2, 9].

As one alternative to tobacco smoking, electronic cigarettes (e-cigs) have gained in popularity and are becoming widely used. There have been some claims that e-cigs may be the cornerstone of a harm reduction strategy against smoking. Indeed, it has been shown that e-cigs may be more attractive and cheaper than other nicotine replacement therapies; they may facilitate smoking cessation, reduction, or abstinence, and some published studies concluded that there is no current evidence of undesirable uptake from nonsmokers [10–12]. Furthermore, e-cigs may pose only a small fraction of the risks of tobacco cigarettes (as tested liquids and aerosols contain negligible concentrations of toxicants and carcinogens, and use of nicotine without tobacco toxicants may pose little risks for the majority of the population) [13–16]. Varlet et al. [15] demonstrated for instance that the oral acute toxicity of e-liquids seems to be of minor concern, and Farsalinos et al. [16] added that overall exposure to metals from e-cigs use may not be of significant health concern for smokers switching to e-cigs.

On the other hand and intrigued by the rapid augmentation of e-cigs use to levels higher than cigarette smoking which declined significantly among US youths [17, 18], Warner demonstrated that nonsmoking youths were unlikely to use e-cigs; most of those who did so used e-cigs only on 1-2 of the past 30 days, hence just experimenting the devices [19]. It was shown by contrast that pupils who had tried smoking, who used to smoke, or who are current smokers were more likely to have used e-cigs and on many more days [19, 20]. A deeper analysis revealed that only about one-fifth of adolescents used nicotine-containing vaporizers, most of them using flavouring-vaporizers, inferring that the rise in adolescent vaporizer use does not necessarily indicate a nicotine epidemic [21].

In low- and middle-income areas where the burden of tobacco smoking is the highest and still fast increasing [2, 22], such as in sub-Saharan Africa (SSA), a key question warrants an urgent and thorough reflection, to know whether it is preferable for these regions to have so many people smoking, or they should be provided with an additional tool (supplementing other methods) to reduce or quit smoking: e-cigs, for instance. In the absence of studies addressing e-cig safety and toxicity in SSA, the present review attempts to see whether e-cigs implementation or vulgarization in SSA countries could be helpful in curbing the toll of tobacco smoking in the region, after revisiting the current burden of tobacco smoking in SSA, the evidence on e-cig safety and toxicity in comparison with conventional tobacco, and the current tobacco control policies in the region.

## 2. Increasing Trends of Tobacco Smoking and Consequential Augmentation in Smoking-Related Diseases in Sub-Saharan Africa

There are about 1.3 billion smokers worldwide, with nearly 80% of them living in low- and middle-income countries (LMIC) where the burden of tobacco-related illnesses and deaths is the heaviest [2, 22, 23]. Besides, tobacco use accounts for 7% of all female and 12% of all male deaths globally [2].

In 2010, the estimated prevalence of smoking in SSA was 14% in males and 2% in females [24]. According to 2012 WHO estimates for the African region, this prevalence was almost at 22% in males and 2-3% in females, hence an overall prevalence around 12% [2]. A recent systematic review conducted by Brathwaite et al. who compiled data between 2007 and 2014 from 13 SSA countries (mostly from Eastern, Western, and Southern Africa) showed that the prevalence of smoking varied immensely across SSA, from 1.8% to 25.8% [25].

It is worth highlighting therefore that the prevalence of tobacco use is rapidly increasing in Africa [5, 25, 26]. Indeed, we learn from Brathwaite et al. that cigarette consumption in Africa and some Middle Eastern countries increased by 57% between 1990 and 2009 while it decreased by approximately 26% in Western Europe during the same period [25]. It is anticipated that the African smoking prevalence will rise to 22% by 2030 if nothing is done [27]. It has been suggested that the increase in tobacco smoking in the African region may be fuelled by increased disposable income along with adoption of Western lifestyles (driven by images such as films that portray smoking as a stylish activity) and increased marketing by tobacco companies [28].

There is sufficient evidence pointing tobacco smoking as a main risk factor for CVD, hypertension, and diabetes mellitus in SSA [29–32]. Moreover, Kruger et al. showed early vascular changes in young normotensive black Africans exposed to cigarette smoking alongside related oxidative stress, hence augmenting their vulnerability to develop early onset arterial stiffness [33].

Furthermore, chronic obstructive pulmonary disease (COPD) has become an increasing health threat in SSA, mainly driven by tobacco smoking and exposure to biomass fuels. It is predicted that, by 2025, COPD will become the third leading cause of death in Africa, surpassing HIV infection [8]. On the other hand, in SSA like in other regions of the globe, smoking has been found associated with a number of cancers, with the first being lung cancer. In fact, smoking accounted for 65% of lung cancer cases in South Africa [7]. We read from Islami et al. that the proportion of lung cancer deaths attributable to smoking approaches 40% in SSA [5]. These authors predicted a likely increase in lung cancer mortality across SSA if appropriate tobacco control programs are not put in place [5]. Tobacco smoking was also found to be the major risk factor for other cancers such as oesophageal, oral cavity, and laryngeal cancers among black Africans [6].

Overall, it is unsurprising that the increase in tobacco smoking in SSA has been accompanied by a concomitant increase in the burden of NCDs all around the region [1, 2]. Moreover, smoking-related illnesses cost billions of dollars each year, imposing a heavy economic toll on countries, in terms of both direct medical care for adults and lost productivity [2]. In SSA like elsewhere, tobacco users who die prematurely deprive their families of income, raise the cost of healthcare, and hinder economic development [34].

## 3. Current Status of Tobacco Control Policies in Africa

Blecher and Ross deplored that tobacco use has received little attention in Africa, mainly explained by the perceived low prevalence of smoking in the region contrasting with high prevalence rates in other developing regions; this has led to a low priority for tobacco control in the continent, ignoring that Africa presents the greatest threats in terms of future smoking expansion [27]. Indeed, looking at the status of tobacco control legislations in Africa, Tumwine showed that African countries are still very far from full implementation of the WHO Framework Convention on Tobacco Control guidelines, especially regarding protection from exposure to tobacco smoke, packaging and labelling of tobacco products, and tobacco advertising, promotion, and sponsorship [35]. For the specific case of West African countries, it was recently shown that tobacco policy interventions remain mostly at a middle or low level of implementation [36], perhaps translatable to the rest of the SSA region. It is true nevertheless that some countries like South Africa have made a lot of efforts, which have resulted in decreasing the country smoking prevalence from almost 33% in 1993 to 16.4% in 2012 [27, 37].

Hence, there is a crucial need for African countries to adopt and implement and/or enforce comprehensive tobacco control strategies proven to be effective in other parts of the world, in order to discount the fast and continuously increasing burden of tobacco smoking in the continent. Indeed, a recent modelling analysis bolstered the urgent need for a more ambitious approach to tobacco control/reduction which will contribute the most in decreasing premature NCD mortality in SSA [38]. In this regard, there have been some claims that e-cigs could be of substantial help in attaining such goals [10–12]. But whether e-cigs are a safer substitute of conventional tobacco is an issue warranting a clear examination.

## 4. Are Electronic Cigarettes Risk-Free?

### 4.1. Effects on Health

Compared to traditional tobacco, e-cigs have no deleterious effect on systolic blood pressure and on systolic and diastolic ventricular functions as well [39]. Although acute smoking causes a delay in myocardial relaxation, e-cigs use has no immediate effects in this concern [39]. However, such as traditional tobacco but at a lower level, e-cigs significantly increase diastolic blood pressure [39], as well as heart rate after 5–10 minutes of use [40, 41], though these transient blood pressure and heart rate elevations have no effects on long-term cardiovascular prognosis. Moreover, the effect of e-cigs on lipid metabolism remains unknown. Therefore, it is currently possible to conclude that e-cigs may be significantly less dangerous for the heart and blood vessels than traditional tobacco, though there is need for further studies in order to underpin this conclusion with robust evidence, especially in SSA.

White blood cells, lymphocytes, and granulocytes counts seem to be insignificantly modified by e-cigs use compared to traditional tobacco use [42, 43]. Furthermore, there is no definite effect of e-cigs on carbon monoxide plasma levels, as these devices do not emit any carbon monoxide [40, 44].

Concerning the respiratory system, e-cigs increase respiratory impedances (a marker of peripheral airway flow resistance) and airway resistance [45, 46]. They can cause irritation of the mouth, pharynx, and upper and lower respiratory organs; they can also cause dry cough [47, 48]. The glycol and glycerol vapours and mist components of e-cigs are known to cause dry mucous membranes [47]. Additionally, the use of e-cigs has been found associated with nose bleeding, change in bronchial gene expression, release of cytokines and proinflammatory mediators, and increase in allergic airway inflammation which can exacerbate asthmatic symptoms, thus by elevating infiltration of inflammatory cells including eosinophils into airways [49–51]. Notwithstanding, all these effects may be lower than those caused by traditional tobacco [52].

It is known to date that traditional tobacco is involved in 80–90% of COPD occurrence [53]. Traditional tobacco smoking has been shown to reduce the level of fractional exhaled nitric oxide (FeNO), a marker for COPD, both acutely and on long-term basis [54]. Likewise, Meo and Al Alsiri highlighted that e-cigs can lead to decreased FeNO synthesis in the lungs [51]. However, lung cells exposure to e-cig vapours results in a far less toxicity than exposure to conventional tobacco [55]. Although it is not clear whether long-term use of e-cigs could lead to COPD, the previous observations permit to infer that current smokers may have a much lower risk of developing COPD if they are switched to e-cigs while nonsmokers who start using e-cigs may present a slight increase in the risk of developing COPD compared to not using any inhalational habit. Studies are nonetheless needed to investigate the relation between e-cigs use and COPD.

Moreover, recent evidence has suggested valuable improvements in asthma outcomes among asthmatic smokers who have substantially reduced their tobacco consumption by switching to e-cigs. In fact, Polosa et al. observed significant and stable improvements in respiratory symptoms, lung function, asthma outcomes, and tobacco consumption in e-cig users with asthma, with no significant changes in exacerbation rates; further, these authors showed that these beneficial effects may persist in the long term [56].

The long-term effect of e-cigs use with regard to the risk of developing cancer remains a topic of debate. Meo and Al Alsiri suggested that e-cigs use may be a risk factor for lung cancer [51]. However, while e-cig users can be exposed to known carcinogens, it seems that their toxicity may be much less than that from traditional tobacco. One study compared the aerosol generated from 12 brands of e-cigs with regular cigarette smoke. The e-cig aerosol contained lower levels of toxicants compared with conventional cigarettes. Carcinogen levels were 9 to 450 times lower than those in conventional tobacco products [57]. On the contrary, a more recent study utilizing the newer “tank-style” systems with higher voltage batteries reported that these e-cigs might expose users to equal or even greater levels of carcinogenic formaldehyde than tobacco smoke [58]. But this study was criticized, for having used unrealistic patterns which resulted in extreme overheating [59]. Furthermore, Farsalinos et al. demonstrated that, under normal vaping conditions, aldehyde emissions are minimal, even in new-generation high-power e-cigs [60].

### 4.2. Toxicity of E-Cig Constituents

The liquid composition of each brand of e-cigs may differ, making it difficult to generalize about the potential toxic properties of these devices. While Schroeder and Hoffman showed in their systematic review that e-cigs deliver less nicotine per puff than regular cigarettes [61], Farsalinos et al. demonstrated that e-cigs that use tank-type atomizers appear to deliver nicotine in equal or more consistent quantities than tobacco cigarette [62]. However, the smoking style does affect the actual nicotine delivered. Indeed, inexperienced e-cig smokers tend to achieve lower serum nicotine concentrations than experienced e-cig smokers who may achieve systemic concentrations similar to those from regular cigarettes [61]. E-cigs have a liquid reservoir of concentrated nicotine: intentional and unintentional exposures from the ingestion of this liquid have been reported [63]. Nicotine is rapidly absorbed in lungs but slowly absorbed from the skin, from mucous membranes, and it undergoes first pass metabolism in the liver [55, 63]. Some estimates suggest that the minimal limit capable of causing fatal outcomes is 0.5–1 g of ingested nicotine, which corresponds to an oral LD_50_ device of 6.5–13 mg/kg [64].

Moreover, Varlet et al. [15] examined 42 models from 14 brands of refill liquids for e-cigs in order to assess the presence of microorganisms and toxic products. All the liquids under scrutiny complied with norms for the absence of microorganisms [15]. None of the products assessed were totally exempt of potentially toxic compounds. However, for products other than nicotine, the oral acute toxicity of the e-liquids tested seemed to be of minor concern [15]. Contrariwise, a minority of liquids, especially those with flavourings, showed particularly high ranges of chemicals, causing concerns about their potential toxicity in case of chronic oral exposure [15]. Concurring with Varlet et al.'s results, Farsalinos et al. showed that overall exposure to metals from e-cigs use is not expected to be of significant health concern for smokers switching to e-cigs use, though it is an unnecessary source of exposure for never-smokers [16]. By contrast to regular cigarettes which are in constant combustion when used, e-cigs release aerosols only when the user exhales. It has been reported that consumption of e-cigs causes aerosol and nicotine emissions into air but at a lower level than conventional cigarettes [65–67]. This suggests thus that e-cigs may have a lower devastating effect with regard to second-hand smoking in comparison with traditional cigarettes.

### 4.3. Smoking Cessation

Results of studies are contradictory concerning the effect of e-cigs on smoking cessation. One trial including 657 subjects found that, at 6 months, the verified abstinence rates were 7.3%, 5.8%, and 4.1%, respectively, with nicotine-containing e-cigs, nicotine patches, and placebo e-cigs. This study did not demonstrate any superiority of nicotine e-cigs compared with other treatments [68]. Likewise, a study among smoking cancer patients referred to a tobacco cessation program reported that e-cig users were twice as likely to be smoking at follow-up as compared with nonusers, after adjusting nicotine dependence, quit attempts, and cancer diagnosis. In this study, e-cig users were more nicotine dependent [69].

The ECLAT trial included 300 tobacco smokers who did not intend to quit. They were divided into 3 groups: 2 were offered different nicotine concentrations in e-cigs, in comparison to a third placebo group. Contrariwise to the former studies, authors of the ECLAT trial concluded that the use of e-cigs, with or without nicotine, decreased cigarette consumption and facilitated sustained tobacco abstinence without causing significant side effects [70, 71]. A randomised crossover trial conducted among 40 adults and dependent smokers of 10 or more cigarettes per day demonstrated that e-cigs alleviated desire to smoke after overnight abstinence and were well tolerated more like the Nicorette inhalator than a tobacco cigarette [72]. Another small trial including 86 smokers concluded alleviated desire to smoke [73].

A longitudinal study in 2 US metropolitan areas including 695 smokers concluded that daily use of e-cigs for at least 1 month is 6 times associated with quitting smoking [74]. A large cross-sectional survey was conducted including 5,863 English adults who had smoked within the previous 12 months and made at least one quit attempt during that period with either an e-cig only (*n* = 464), nicotine replacement therapy bought over-the-counter only (*n* = 1,922), or no aid in their most recent quit attempt (*n* = 3,477). This study concluded that, among smokers who have attempted to stop without professional support, those who used e-cigs were more likely to report continued abstinence than those who used a licensed nicotine replacement therapy product bought over-the-counter, or no aid to cessation [75]. A large online study including 19,414 e-cig users with a median use of 10 months reported complete substitution of smoking (from conventional tobacco to e-cigs) by 81% of participants while current tobacco smokers had reduced smoking consumption from 20 to 4 cigarettes per day [76]. In a study of 27,460 European Union adult citizens, 35.1% and 32.2% of participants reported smoking cessation and reduction with the help of e-cigs, respectively [77]. Eventually, a large Korean Web-based survey showed that participants who had made an attempt to quit were more likely to use e-cigs [78].

It is worth noticing however that some studies have yielded contrary results. Indeed, in a longitudinal study among US smokers, e-cigs may not increase rates of smoking cessation [79]. Other studies in US and England reported that the use of e-cigs may be at increased risk for not being able to quit smoking [80, 81].

Overall, despite the discrepant evidence with respect to e-cigs effectiveness towards smoking cessation, one can observe that some authors have demonstrated that e-cigs may favour smoking cessation, though data remain controversial to date. A recent systematic review concluded that e-cigs may help smokers to stop smoking in the long term, though the small number of trials found eligible for this review led to a low rated confidence in these results [48]. Nonetheless, at least 27 studies are ongoing, which will hopefully demystify the relation between e-cigs and smoking cessation, reduction, or abstinence, as well as the long-term toxic effects of e-cigs.

## 5. Could E-Cigarettes Help to Curb the Increasing Tobacco-Driven Burden of Noncommunicable Diseases in Sub-Saharan Africa?

In response to the growing epidemic of tobacco smoking, WHO introduced in 2008 six MPOWER measures to reduce the burden of this disastrous health threat without the effective implementation of which tobacco could be responsible for over 1 billion deaths in this century, mostly in LMIC where cessation is uncommon [2, 9, 22]. Two of these measures are protecting people from second-hand smoking and offering help to quit tobacco use [2, 9]. On the basis that e-cigs may facilitate smoking cessation, reduction, or abstinence [10–12, 70–76, 78, 82] and that they may pose only a small fraction of the risks of tobacco cigarettes [13–16, 39, 52, 57, 61, 63, 65–67], it is likely that e-cigs may address the just-cited 2 MPOWER measures, hence contributing to the reduction in the heavy toll driven by tobacco smoking.

Accordingly, a recent independent report commissioned by the Public Health England concluded that e-cigs are 95% less harmful than tobacco and that when supported by a smoking cessation service, they help most smokers quit tobacco altogether [83]. This conclusion was drawn from the following facts: (i) constituents of cigarette smoke that harm health are either absent in e-cig vapour or, if present, are mostly at levels much inferior to 5% of smoking doses; (ii) the main chemicals present in e-cigs only have not yet been linked with any serious risk [84].

In SSA like in other low- and middle-income settings where the burden of tobacco-related illness and death is the heaviest [2, 22, 23] with consequential increase in the burden of NCDs, there is urgent need to identify and implement all measures that could help to curb the burden of tobacco smoking. According to Jha and Peto [22], widespread cessation of smoking is the most important way to help achieve this goal. But either for genetic or constitutional reasons or due to environmental and behavioural challenges, many smokers build an extensive history of failed quit attempts [85, 86]. In this context, a reasonable secondary tobacco control approach could be to try and reduce the harm from continued tobacco use amongst smokers unable or unwilling to quit. Possible approaches to reduce the exposure to toxins from smoking include reducing the amount of tobacco used and using less toxic products, such as e-cigs [87]. In fact, considering the potentially less harmful effects of e-cigs [10–12, 70–78, 82, 83], switching from conventional tobacco to e-cig use may help to fight against the devastating effects of tobacco smoking. Besides, e-cigs may not cause second-hand smoking; if so, this effect might be much lower than that from conventional tobacco [15, 16, 65–67]. Some experts have additionally presented e-cigs as a means to reduce the health costs of combustible tobacco use which, as shown above, is highly enormous in SSA [34, 85].

However, it is worth fearing that wide-scale promotion and use of e-cigs, fuelled by an increase in the advertising of these products, may carry substantial public health risks [85]. Indeed, nonsmokers may start using e-cigs because they have heard it is less harmful than traditional tobacco rather than remaining naïve of smoking which is by far the best attitude. Besides, e-cigs may serve as a gateway product, that young people who first experiment with these products will move on to traditional tobacco use. Further, normalization of e-cig use may lead former cigarette smokers to begin using this new device, thereby reinstating their nicotine dependence and fostering a return to tobacco use [85]. Nevertheless, evidence in the adult population shows that e-cigs appear to be largely confined to current or former smokers, while current use and nicotine use by those who have never smoked seem rare [77]. The situation is even better among the youths, where e-cigs use is mostly experimentation and use of nonnicotine devices [19–21, 78]. Definitely, although the fear seems unjustified, e-cigs use needs to be continuously monitored on a population level, considering that there is not yet enough evidence pointing e-cigs use as risk-free, especially if used in the long term.

Implementation and vulgarization of e-cig use in SSA must therefore be conducted with caution and controlled and regulated to avoid possible disastrous long-term effects. In fact, there is no regulation in the marketing, use, and consumption of e-cigs [63]. Insufficient regulation might contribute to the expansion of the e-cig market, in which tobacco companies have a substantial stake, potentially renormalizing smoking habits and negating years of intense campaigns against conventional tobacco use. WHO is currently working with national regulators to investigate the various regulatory options and with toxicologists to better understand the possible effects of e-cigs on health [88]. A document in this regard is expected to be released soon. Nonetheless, one should bear in mind that excessive regulation should be avoided because it could marginalize e-cigs in favour of traditional tobacco smoking.

For now, as it is the case for traditional tobacco [2, 9], advertisements on e-cigs should be prohibited. Further, e-cigs should be strictly recommended to smokers and/or ex-smokers only, as a method to quit smoking or prevent relapse, and never-smokers should be strongly encouraged not to use these devices. Sales of e-cigs to young SSA populations should be banned in order to avoid use by unintended populations.

It is guessable that if e-cigs are introduced in SSA, the number of persons on current tobacco smoking will gradually drop down in favour of e-cigs as seen elsewhere [17, 18], with a consequential and concomitant decreased tobacco-related burden in the region. Second-hand smoking may also diminish considerably, in a context where people smoke everywhere without any fear or control. However, no study has already been conducted in SSA to assess the efficacy and safety of e-cigs use with respect to tobacco smoking cessation, reduction, or abstinence, and this gap must be filled. Well-designed studies are also needed to measure the toxicity and environmental impact associated with the use of e-cigs in SSA. On the other hand and considering that the large majority of people in the region are of low socioeconomic background, it is questionable whether e-cig implementation in SSA could be cost-beneficial. Another issue will be to make e-cigs available everywhere in the region, especially in remote areas.

## 6. Conclusions

Clearly, the high and continuously increasing burden of NCDs in SSA may be in great part explained by the increasing burden of tobacco smoking in the region. Therefore, reduction in the burden of tobacco smoking could result in the substantial curbing of NCDs in SSA, especially through smoking cessation. E-cigs may facilitate smoking reduction, cessation, or abstinence and may be less toxic than traditional tobacco; however, studies are needed in SSA to better investigate these issues in the region. Actually, if introduced, e-cigs use in SSA should be strictly recommended to current and/or ex-smokers only, and nonsmokers should be discouraged from any temptation. Advertisements should be prohibited and young populations should not have access to these devices. Their usage must be continuously controlled and monitored, given that they may not be risk-free, especially if used for long.

## List of Abbreviations

CVD: Cardiovascular disease
COPD: Chronic obstructive pulmonary disease
E-cig: Electronic cigarette
FeNO: Fractional exhaled nitric oxide
NCDs: Noncommunicable diseases
SSA: Sub-Saharan Africa
WHO: World Health Organization.
